# Carbon neutrality: Toward a sustainable future

**DOI:** 10.1016/j.xinn.2021.100127

**Published:** 2021-06-06

**Authors:** Jing M. Chen

**Affiliations:** 1 University of Toronto, Toronto, ON, Canada; 2 Fujian Normal University, Fuzhou, Fujian, China

Carbon neutrality refers to net-zero carbon dioxide (CO_2_) emissions attained by balancing the emission of CO_2_ with its removal so as to stop its increase in the atmosphere that causes global warming. As of February 2021, 124 countries had pledged to achieve carbon neutrality by 2050 or 2060. This is a remarkable development reached after the annual United Nations Conference of the Parties of 1995, in particular those of Kyoto (1997), Bonn (2001), Bali (2007), and Paris (2015), with progressively more concrete binding commitments to emission reduction by the parties (countries).

By 2020, global average atmospheric CO_2_ concentration had reached 415 ppm, a large increase from its preindustrial level of 285 ppm around 1850. As a result, the global average surface temperature increased by about 1.2°C over the period 1850–2020.^1^ As the additional CO_2_ in the atmosphere continues to produce a greenhouse effect, the Earth is committed to further warming, even if we stop carbon emissions immediately. The goal of carbon neutrality by 2050 is to limit the temperature increase by 2100 to 1.5°C–2.0°C from its preindustrial level.^2^ Enormous efforts by all countries are needed to achieve this goal. These could be regarded as desperate efforts in the face of dangerous climate change that may even threaten the very existence of our species on Earth. The warming in the recent past has already damaged our living environment on a gigantic scale, and the list is already long: insects, drought, flood, wildfires, species extinction, loss of biodiversity, ocean acidification, glacier retreat, Arctic and Antarctic ice melt, sea-level rise, etc. In my view, sea-level rise is a particularly serious issue that could potentially threaten over 100 million people in this century and much more in longer terms. In Earth’s history, the sea level has varied by about 200 m, while temperature varied by about 10°C, i.e., the sensitivity is 20 m per °C.^3^ In the Eocene, about 40 million years ago, the Earth’s surface temperature was about 3.5°C warmer than the present temperature and the sea level was about 75 m higher than the current sea level; at the last glaciers’ maximum about 20,000 years ago, when the temperature was about 6°C lower, the sea level was about 125 m lower. Although the Earth’s surface temperature has risen by 1.2°C since the preindustrial period, the sea level rise has been 0.24 m, and the projected rise by 2100 is in the range of 0.3–1.5 m, depending on the fossil fuel emission scenario.^4^ It will take thousands of years for the sea level to increase to its potential height at a given temperature because it is a slow process to warm the oceans, which have an average depth of about 3,600 m, to reach the maximum sea ice melt and seawater thermal expansion. Therefore, we would expect that the sea level would continue to rise even if the increase in CO_2_ concentration in the atmosphere is stopped by 2050, as the existing CO_2_ and other greenhouse gases will continue to add more heat to the Earth’s system. The only way we can stop the gradual and long-term rise of the sea level is to reduce atmospheric CO_2_ to close to the preindustrial level. This would require more than achieving carbon neutrality, meaning that we need not only to balance carbon emissions with removals, such as carbon sinks in ecosystems, but also to have removals larger than emissions. Nevertheless, carbon neutrality would be a giant first step of humankind in stopping the accelerated damage to our living environment.

The internationally concerted effort toward carbon neutrality could be the largest international agreement achieved in human history. This is a positive sign of international societal development but could also be regarded as an act of desperation to protect ourselves from damages caused by ourselves. We have wasted much time in realizing the seriousness of the global warming issue and in taking necessary actions to address the issue since the expression of the first consensus view among multi-national scientists in the First Assessment Report of the Intergovernmental Panel for Climate Change in 1992. We should now be desperate in taking actions not only to curb carbon emissions but also to find solutions to the energy crisis. In the short span of time since 1850, we have depleted nearly half of fossil fuel resources^5^ that took hundreds of millions of years to form throughout the entire Earth’s history, and at the current rate of exploitation, oil and natural gas may last for only 40–80 years and coal for about 100 years. It is obvious that the current fossil energy consumption is not sustainable. Therefore, we should also be desperate in finding ways to address the energy crisis. Carbon neutrality would be the ultimate solution to this crisis.

To achieve carbon neutrality, we first need to reduce carbon emissions in as many ways as possible, including (1) replacing fossil fuels with carbon-free renewable energies, hydropower, and nuclear power; (2) industrial CO_2_ capture, removal, storage, and utilization; (3) reuse of solid wastes; and (4) reducing energy consumption and increasing energy use efficiency. In the meantime, we should also enhance carbon sinks in land and ocean. The potentials of renewable energies, including wind, solar, biomass, geothermal, tidal, and hydrogen energies, are enormous and can entirely satisfy our energy needs. As technologies develop, it is hoped that these energy sources could become as cheap as fossil fuels, or their costs may soon be lowered to below the sum of the fossil fuel cost and the social cost of carbon, which is recently pegged at US$52/tCO_2_ by the US federal government. In other words, if the international society concertedly takes global warming as a serious issue and uses a pertinent high price for carbon based on evaluations of the potential damage of carbon emission to the Earth’s environment, it would provide a strong economic incentive to develop renewable energies, and our society would move in the right direction toward a carbon-free future. Enhancing carbon sinks in land could initially be a low-cost option for carbon removal from the atmosphere, as tree planting and forest management can remove carbon at much lower costs than industrial carbon removal. Maximizing land sinks, therefore, should be a priority in our agenda to achieve carbon neutrality in the near future, especially where such potentials remain high. However, land sinks have limits and the sequestered carbon in biomass and soil is not permanently safe from returning to the atmosphere, so we would consider land sinks as an option to buy time in curbing the net carbon emissions to the atmosphere. There is also a large potential to use land to “farm” carbon from the atmosphere, i.e., to grow biomass and use it as a source of energy to replace fossil fuels. In our drive toward carbon neutrality, biomass energy could play a continuous and important role. It may not be possible to reach carbon neutrality without industrial carbon capture, removal, and storage, because we will continue to depend on fossil fuels to some extent in the near future. When carbon markets are established with high carbon prices, technologies and infrastructures for implementing these industrial options to reduce emissions could be encouraged to develop and eventually play a dominant role in achieving carbon neutrality. Energy-conserving lifestyles should also be encouraged.

**Figure 1:**
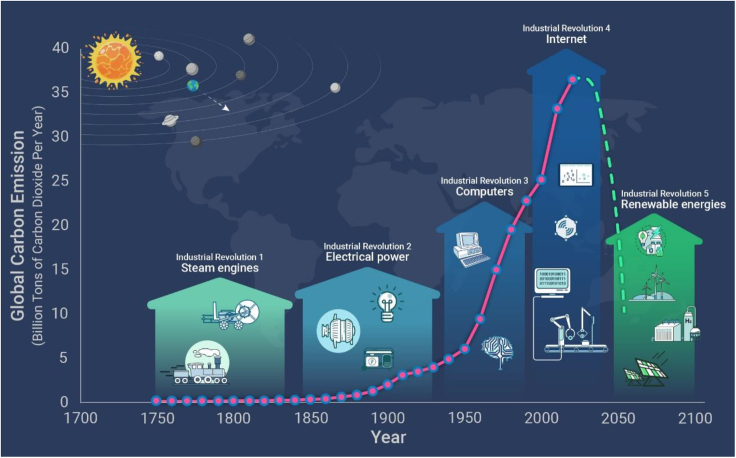
Carbon neutrality may be regarded as the fifth industrial revolution

Carbon neutrality will greatly slow down global warming and solve our energy crisis, with accompanying benefits to air quality, ecological recovery, and landscape beautification. It may, therefore, be regarded as an industrial revolution that would mark an important milestone in human development. Following the previous four industrial revolutions, carbon neutrality could be the fifth (Figure 1). The first occurred around 1750 and accelerated after successful operation of steam engines designed by James Watt in 1785, which powered large-scale industries. The second took shape around 1850, when the discovery of electricity by Benjamin Franklin in 1732 led to widespread use of electrically powered machines and production lines that greatly improved industrial productivity. The third came shortly after the first computer produced by John Mauchly and Presper Eckert in 1946, which made automatic production and other industrial processes possible. The fourth emerged gradually, after the formation of the first worldwide web in 1983, and at the turn of this century it propelled a digital era with the internet of things + artificial intelligence + big data that allowed for efficient production and distribution of goods and customized services and thus greatly improved the well-being of everyone. These industrial revolutions in sequence improved our living standards at the expense of natural resources of various types, many of which are not renewable. At the core of the issues in the relationship between humans and nature is our consumption of fossil fuels, which causes not only global warming but also the degradation of our environment. The fifth industrial revolution could solve these core issues, and, therefore, carbon neutrality would be the first step toward a sustainable future in which humans and nature can harmoniously coexist.
